# Bedaquiline exposure in pregnancy and breastfeeding in women with rifampicin‐resistant tuberculosis

**DOI:** 10.1111/bcp.15380

**Published:** 2022-05-26

**Authors:** Richard Court, Kamunkhwala Gausi, Buyisile Mkhize, Lubbe Wiesner, Catriona Waitt, Helen McIlleron, Gary Maartens, Paolo Denti, Marian Loveday

**Affiliations:** ^1^ Division of Clinical Pharmacology, Department of Medicine University of Cape Town; ^2^ Wellcome Centre for Infectious Diseases Research in Africa (CIDRI‐Africa), Institute of Infectious Disease and Molecular Medicine University of Cape Town Cape Town; ^3^ Department of Pharmacology and Therapeutics University of Liverpool UK; ^4^ HIV Prevention Research Unit, South African Medical Research Council KwaZulu‐Natal South Africa; ^5^ CAPRISA‐MRC HIV‐TB Pathogenesis and Treatment Research Unit University of KwaZulu‐Natal South Africa

**Keywords:** breastfeeding, pharmacokinetics, pregnancy

## Abstract

**Aims:**

We aimed to explore the effect of pregnancy on bedaquiline pharmacokinetics (PK) and describe bedaquiline exposure in the breast milk of mothers treated for rifampicin‐resistant tuberculosis (TB), where there are no human data available.

**Methods:**

We performed a longitudinal PK study in pregnant women treated for rifampicin‐resistant TB to explore the effect of pregnancy on bedaquiline exposure. Pharmacokinetic sampling was performed at 4 time‐points over 6 hours in the third trimester, and again at approximately 6 weeks postpartum. We obtained serial breast milk samples from breastfeeding mothers, and a single plasma sample taken from breastfed and nonbreastfed infants to assess bedaquiline exposure. We used liquid chromatography–tandem mass spectrometry to perform the breast milk and plasma bedaquiline assays, and population PK modelling to interpret the bedaquiline concentrations.

**Results:**

We recruited 13 women, 6 of whom completed the ante‐ and postpartum PK sampling. All participants were HIV‐positive on antiretroviral therapy. We observed lower ante‐ and postpartum bedaquiline exposures than reported in nonpregnant controls. Bedaquiline concentrations in breast milk were higher than maternal plasma (milk to maternal plasma ratio: 14:1). A single random plasma bedaquiline and M2 concentration was available in 4 infants (median age: 6.5 wk): concentrations in the 1 breastfed infant were similar to maternal plasma concentrations; concentrations in the 3 nonbreastfed infants were detectable but lower than maternal plasma concentrations.

**Conclusion:**

We report low exposure of bedaquiline in pregnant women treated for rifampicin‐resistant TB. Bedaquiline significantly accumulates in breast milk; breastfed infants receive mg/kg doses of bedaquiline equivalent to maternal doses.

**What is already known about this subject**

- The effect of pregnancy on bedaquiline pharmacokinetics is unknown.
- There are no human data of bedaquiline exposure in breast milk, and subsequent exposure to breastfeeding infants.
- A previous animal study described bedaquiline concentrations in rat milk to be 6–8‐fold higher than maternal plasma concentrations.

**What this study adds**

- We describe lower antepartum exposures of bedaquiline compared to nonpregnant patients.
- There was no difference between ante‐ and postpartum bedaquiline pharmacokinetics.
- We observed high concentrations of bedaquiline in breast milk with a milk:plasma ratio of 14:1.
- The 1 infant breastfeeding had plasma bedaquiline concentrations similar to maternal plasma.

## 1 INTRODUCTION

Acquisition of data quantifying the exposure of second‐line tuberculosis (TB) drugs in pregnant woman treated for rifampicin‐resistant TB (RR‐TB) is a priority. Until recently, pregnant and breastfeeding women have typically been excluded from clinical trials of new drugs, including TB treatment.^1^ The World Health Organization currently recommends individualised treatment regimens with drugs with a preferred safety profile for pregnant women with RR‐TB,^2^ although there are limited human data guiding these recommendations.

Bedaquiline is a group A drug, recommended for inclusion in all RR‐TB treatment regimens, and, although used in pregnant women, safety data are lacking. The design of pharmacokinetic (PK) studies to explore the effect of pregnancy on long half‐life drugs like bedaquiline is challenging, as cumulative drug concentrations could mask pregnancy‐related effects on drug exposure. Physiological changes in pregnancy result in decreased concentrations of many drugs, particularly in the third trimester.^3^ PK in pregnancy may be complex; 1 of the reasons for reduced drug concentrations in pregnancy is the reduction in plasma concentrations of the 2 key drug‐binding proteins, albumin and α1‐acid glycoprotein.^4^ Reduction in these binding protein concentrations reduces the total (bound and unbound) concentrations of drugs, but the unbound fraction typically increases, resulting in unbound drug concentrations that are similar to nonpregnant women. As only the unbound drug is pharmacologically active, recommendations to increase the dose of drugs in pregnancy to approximate total concentrations in nonpregnant women could therefore increase the risk of toxicity. However, pregnancy also increases several drug clearance mechanisms, which could reduce unbound drug concentrations. Although measurement of unbound bedaquiline concentrations is preferable to rationally optimise dosing in pregnancy (bedaquiline is >99% protein‐bound),^5^ an understanding of the effect of pregnancy on total bedaquiline concentrations would provide a much‐needed foundation to understand the effect of pregnancy on the unbound fraction.

Data on the secretion of key drugs for RR‐TB into breast milk are scarce. The studies describing RR‐TB drug exposure in breast milk are small with few or no infant plasma PK data available—the study designs are also unclear or unstated. Linezolid,^6^ levofloxacin^7^ and cycloserine^8^ penetrate poorly into breast milk and exposure to breastfed infants is therefore likely to be low. Clofazimine, in contrast, demonstrates effective breast milk penetration with skin discoloration observed in the infants of breastfeeding mothers treated with clofazimine for leprosy^9^, ^10^; clofazimine exposure in breast milk in the context of mothers treated for TB is unfortunately lacking. Animal studies have shown that bedaquiline is concentrated in rat milk with concentrations 6‐ to 12‐ fold higher than maternal rat plasma concentrations,^11^ but there are currently no human data available. Information on clinically relevant infant exposure to RR‐TB drugs through breastfeeding with mother–infant pairs has not been done, and is an important knowledge gap.

An international consensus panel on the inclusion of pregnant and postpartum women in TB drug trials, convened by the NIH, identified the safety, tolerability and PK of novel agents and regimens for treatment of RR‐TB as research priorities.^12^ It is ethically imperative to study drug dosing and safety in populations where drugs are used—this has not been done satisfactorily for RR‐TB.^1^ We therefore conducted an observational study of bedaquiline exposure in pregnant and breastfeeding women with RR‐TB, and explored secondary bedaquiline exposure in their infants.

## 2 METHOD

### 2.1 Study design

We performed a longitudinal PK study in pregnant women aged ≥18 years treated for RR‐TB, and their infants, at King Dinuzulu Hospital (KDH) in Durban, Kwazulu‐Natal—nested within a cohort, which has been previously described.^13^ KDH is a specialist provincial RR‐TB hospital where, until recently, all pregnant women with RR‐TB in Kwazulu‐Natal province were referred for care. With some individual regimen variability, all participants were treated with a minimum of 5 drugs including bedaquiline. Other drugs included: pyrazinamide, isoniazid, clofazimine, linezolid, moxifloxacin, and, less commonly: ethambutol, terizidone, levofloxacin, ethionamide and para‐aminosalicylic acid. We performed PK sampling predose and at 2, 4 and 6 hours postdose in the third trimester of pregnancy (≥28 wk), and at the 6‐week postpartum visit. Dosing on both sampling days was observed after a standard breakfast consisting of a cup of tea/coffee and a peanut‐butter sandwich; the tablets were ingested with 250 mL of water. Considering that bedaquiline is dosed 3 times a week (after the 2‐wk loading dose), it was not always logistically possible to schedule PK sampling on a day when bedaquiline was administered. We therefore recorded the last date and time when bedaquiline was dosed to interpret the exposures with our modelling. The use of concurrent medications, including antiretroviral therapy, and the start date of all TB drugs, including bedaquiline, were recorded. If available, breast milk samples were taken from breastfeeding mothers by manual expression at the same timepoints that blood was drawn at the postpartum visit (i.e. predose, and 2, 4 and 6 h postdose); samples were frozen within 30 minutes of sampling at −80°C. To evaluate infant drug exposure, a single random plasma sample was taken from infants at the postpartum visit. If applicable, the time of the most recent breastfeed prior to the infant blood draw was recorded.

### 2.2 Bedaquiline assays

Plasma and breast milk samples were stored at −80°C and transported to the University of Cape Town, Division of Clinical Pharmacology laboratory where total plasma and breast milk bedaquiline and M2 assays were performed using liquid chromatography with tandem mass spectrometry. The plasma assay for total bedaquiline has previously been described.^14^ Bedaquiline and its M2 metabolite in breast milk were analysed with an assay developed at the Division of Clinical Pharmacology laboratory, validated using Food and Drug Administration and European Medicines Agency guidelines^15^, ^16^; the standards and quality checks were performed using blank donated breast milk. The extraction procedure consisted of protein precipitation and solid phase extraction, followed by gradient liquid chromatography on an Agilent Poroshell 120 SB‐C18 (2.1 mm × 50 mm, 2.7 μm) analytical column with tandem mass spectrometry detection. An AB Sciex API 3000 mass spectrometer at unit resolution in the multiple reaction monitoring mode was used to monitor the transitions of the protonated precursor ions m/z 555.1, m/z 561.1, m/z 541.1 and m/z 545.1 to the product ions m/z 58.2, m/z 64.1, m/z 480.3 and m/z 480.4 for bedaquiline, TMC207‐d6, M2 and M2‐d3C13, respectively. Electro spray ionisation was used for ion production. The calibration curves fitted quadratic (weighted by 1/x) regressions based on peak area ratios over the ranges 0.0780–5.00 μg/mL for bedaquiline and 0.0312–2.00 μg/mL for M2. The combined accuracy (%Nom) and precision (%CV) statistics of the lower limit of quantification, low‐, medium‐ and high‐quality controls of bedaquiline and M2 during intra‐ and intervalidations were between 96.7 and 106.5%, and 3.4 and 7.5%, respectively.

### 2.3 PK modelling

Bedaquiline concentrations were interpreted using population PK modelling in NONMEM version 7.4.5.^17^ Perl‐speaks‐NONMEM version 5.2.6, Pirana version 3.0, and R with the package xpose4 were used to facilitate the model development process, data manipulation and generation of model diagnostics.^18^ As a starting point, we used a published population PK model of bedaquiline in nonpregnant adults with HIV and drug‐resistant TB.^14^ Briefly, the published model consists of 3 disposition compartments for bedaquiline and 1 disposition compartment for M2. There was a correlation between bedaquiline and M2 between‐subject variability on clearance, and residual variabilities. The effect of body weight on all disposition parameters was included using allometric scaling; albumin also affected the drug disposition parameters. The coadministration of ritonavir‐boosted lopinavir reduced bedaquiline and M2 clearance by 65 and 42%, respectively. Molar concentrations were used during model development to account for mass balance between bedaquiline and its metabolite M2. Participant albumin information were not captured in the current study, therefore we imputed a reported albumin concentration from a previous study in South African patients with RR‐TB.^19^

When analysing the data, we first fit the original model as published, without re‐estimating any of the population parameters, but using the study covariate, doses and dosing regimen information. This is similar to using the current data as an *external* validation of the model, i.e. assessing how the previous model predicts the current data based solely on covariate information and assuming no effect of pregnancy (which was not part of the original model). Afterwards, we attempted to use the data to re‐estimate parameter values, using the general principles of model development,^20^ including drops in NONMEM objective function value for assessment of statistical significance and inspection of diagnostic plots. Throughout the modelling process, we assumed 100% treatment adherence unless the participant disclosed otherwise.

### 2.4 Calculation of the milk:plasma ratio

The PK of bedaquiline and M2 in breast milk of the mothers with paired plasma and milk samples was characterised using an effect compartment.^21^ The effect compartment model described an accumulation ratio (milk:plasma, M:P), and a time delay in the equilibration between the breast milk and plasma concentrations.^22^, ^23^ Further information about the effect compartment is provided in the supplementary material.

### 2.5 Calculation of infant bedaquiline intake with breast milk

To estimate how much bedaquiline is ingested per day by a typical child breastfed by a mother receiving bedaquiline, we assumed an average infant milk ingestion of 0.15 L/kg/d.^24^ The following equation was used to calculate the infant dose^25^:

$$D_{\text{infant}}=C_{m}\cdot V_{m}$$

where *Vm* is the volume of milk ingested by breastfeeding and *C*_*m*_ is the drug concentration in breast milk. The *C*_*m*_ was calculated using the formula below:

$$C_{m}=M:P\cdot C_{\text{pavg}}$$

where *C*_*pavg*_ is the average maternal plasma concentration, which will vary depending on the date when participants were initiated on treatment with bedaquiline relative to the date of PK sampling.

### 2.6 Ethics

Ethics approval for the study was granted by the South African Medical Research Council Ethics Committee (EC017–6/2016) and the University of Cape Town Human Research Ethics Committee (HREC: 666/2018). Informed consent was taken from all participants in a language of their choice (either English or isiXhosa).

## 3 RESULTS

### 3.1 Study population and sampling

Bedaquiline PK samples were available from 13 women in the third trimester of pregnancy, at 30 (interquartile range: 25–37) weeks gestation, 6 of whom returned for postpartum sampling at 7 (interquartile range: 6.5–8) weeks after delivery. Seventy‐one plasma samples of bedaquiline parent and metabolite concentration were available for analysis. Participant characteristics are shown in Table 1. All participants were living with HIV and treated with antiretroviral therapy (ART), most commonly with nevirapine‐based ART (*n =* 10, 83.3%); two women were treated with dolutegravir and 1 woman received lopinavir/ritonavir‐based ART. Additional individual participant characteristics, including time on treatment with bedaquiline are shown in Table S1. Serial breast milk samples at the same time‐points that plasma was sampled, were available in 2 breastfeeding participants. A single random plasma bedaquiline concentration was available from 4 infants on the postpartum PK sampling day, of whom 1 was breastfed. The range of gestational age at time of delivery of the 4 infants who had PK sampling was 33–38 weeks. The serial postdose bedaquiline and M2 concentrations at each sampling time point are shown in Table 2.

**TABLE 1:** Characteristics of pregnant women treated for rifampicin‐resistant tuberculosis (TB)

Median (range)	Antepartum (*n =* 13)	Postpartum (*n =* 6)	Infants (*n =* 4)
**Baseline characteristics**			
Age (y)	30 (23–48)	30 (23–48)	6.5 (6–8) wk
Height (cm)	160 (140–176)	162 (152–163)	53 (50–55)
HIV status (pos/neg)	(13/0)	(6/0)	
TB type (RR/MDR/pre‐XDR/XDR/missing)	(6/3/2/1/1)	(2/2/0/1/1)	
Previous TB (yes/no/missing)	(7/5/1)	(2/3/1)	
CD4 (cells/μL)	311 (44–1008)	545 (253–1008)	
ART (NVP/LPV/DTG)	10/1/2	5/0/1	
**Characteristics on the pharmacokinetic sampling day**			
Weight (kg)	61 (55–104)	67(52–84)	4.1 (2.6–7.1)
Time since EFV switch (d)	31 (13–375)	175 (85–421)	
Gestational age/time after delivery (w)	30 (25–37)	7 (6.5–8)	
Inpatients/outpatients	12/1	0/6	
Race (black/white)	13/0	6/0	
Time since BDQ initiation (d)	27 (13–96)	154 (81–201)	

ART, antiretroviral therapy; BDQ, bedaquiline; DTG, dolutegravir; EFV, efavirenz; LPV, lopinavir; MDR, multidrug‐resistant; NVP, nevirapine; RR, rifampicin‐resistant; XDR, extremely drug‐resistant.

**TABLE 2:** Median (range) bedaquiline and metabolite (M2) concentrations per time point*

Time point	Bedaquiline (*n =* 13)				Metabolite, M2 (*n =* 13)			
	Antepartum		Postpartum		Antepartum		Postpartum	
Time	No.	Concentration (mg/L)	No.	Concentration (mg/L)	No.	Concentration (mg/L)	No.	Concentration (mg/L)
Predose	6	0.419 (0.146–0.997)	2	0.186 (0.135–0.237)	6	0.183 (0.0479–0.297	2	0.0584 (0.0440–0.0728)
2 h	6	0.621 (0.225–1.78)	2	0.237 (0.205–0.2678)	6	0.160 (0.0455–0.283)	2	0.0630 (0.0425–0.0834)
4 h	5	1.05 (0.393–1.95)	1	1.06	5	0.144 (0.0469–0.286)	1	0.0467
6 h	5	1.69 (0.296–2.93)	1	1.14	5	0.181 (0.0454–0.285)	1	0.0547
24 h	7	0.308 (0.265 – 0.505)	4	0.3085 (0.227–0.312)	7	0.168 (0.0799–0.281)	4	0.128 (0.106–0.166)
26 h	7	0.250 (0.226 – 0.461)	4	0.281 (0.242–0.293)	7	0.142 (0.0618–0.254)	4	0.107 (0.0989–0.128)
28 h	7	0.228 (0.188 – 0.419)	4	0.275 (0.263–0.296)	7	0.145 (0.0618–0.227)	4	0.112 (0.107–0.132)
30 h	7	0.205 (0.169–0.347)	3	0.284 (0.234–0.299)	7	0.133 (0.0585–0.224)	3	0.111 (0.0970–0.120)

*Time point: approximation of the time after dose; No.: Number of participants at each timepoint.

### 3.2 PK modelling

**FIGURE 1:**
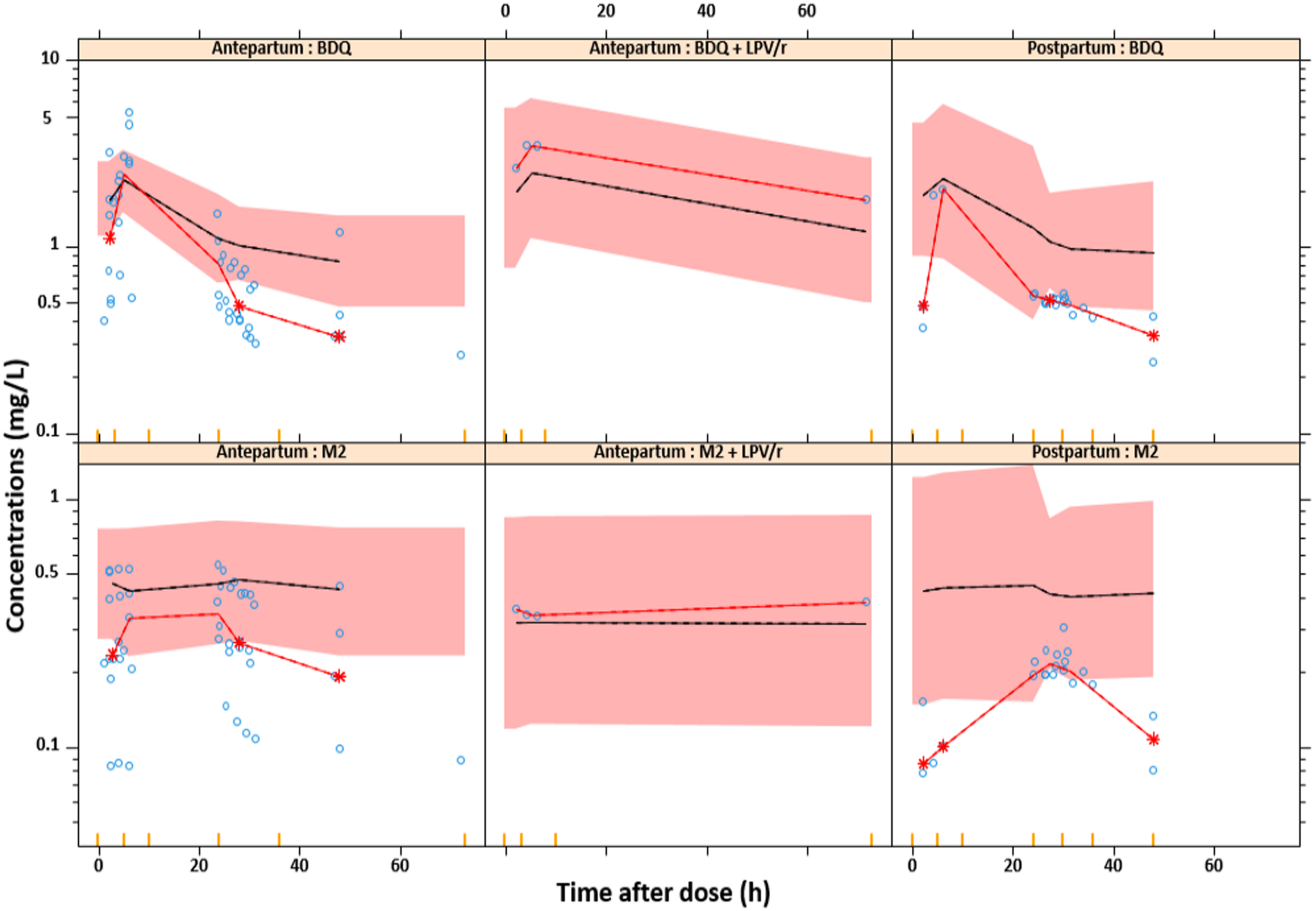
Visual predictive check of the bedaquiline and M2, the top panels represent the parent and the bottom panels represent the metabolite bedaquiline concentrations. The first column displays antepartum concentrations, while the last and middle columns show postpartum concentration and antepartum concentration in the participant coadministered lopinavir/ritonavir, respectively. Due to the small sample size in each panel, we plotted the 50th percentiles of the observations (red line)—the shaded areas represent the 95% model‐predicted confidence intervals and the black line is the median of the model predicted confidence interval

When we used the published model^14^ to predict the expected exposures in these patients (thus using the original population parameter estimates and assuming no effect of pregnancy), the model overpredicted both bedaquiline and M2 concentrations on both antepartum and postpartum visits, as presented in the visual predictive check in Figure 1. The visual predictive check shows that the PK terminal elimination phase of the participant not on lopinavir/ritonavir were approximately 50% lower that the model prediction (for both the metabolite and parent) as illustrated by the deviation of the 50th percentiles of the observations (red line) from the median of the model predicted confidence interval (black line). If the PK parameters in this study were in line with the previous report, we would have expected to observe higher bedaquiline concentrations. Only the data from the participant coadministered lopinavir/ritonavir, who had higher bedaquiline concentrations due to a drug–drug interaction, were in line with the model prediction. The final model PK measures are shown in Table S2. We encountered several challenges when attempting to fit the original model to the current data by re‐estimating the parameter values. The model structure is complex, with multiple disposition compartments, and the current data did not reliably support the re‐estimation of all parameters—some of the parameter estimates obtained when attempting to re‐fit were unstable and/or implausible. In other words, while the model could be adapted to fit the study data, this could be achieved in multiple different ways, e.g. assuming a larger clearance or lower bioavailability (both ante‐ and postpartum) and a larger peripheral volume of distribution. We experienced further complications when trying to estimate a significant difference between the 2 PK sampling visits, i.e. possibly due to pregnancy status. All the scenarios were nearly equivalent in terms of goodness of fit, and there was no meaningful difference in terms of statistical significance, thus leaving the choice largely in the domain of speculation. Choosing a different scenario (on which a difference is ascribed to) would imply a different interpretation of the results, and if the different options for the model were to be used to predict concentrations and suggest dose adjustments they could come to very different conclusions. We also attempted to use a frequentist prior approach^26^ to try and stabilise the parameter estimates, but the results became highly dependent on the assumptions on the prior precision of each parameter, thus not solving the problem. For this reason, we decided to simply use the model as originally published and acknowledge that the concentrations we observed are lower than expected, assuming that the PK are the same as nonpregnant patients.

### 3.3 Breast milk and infant exposures

**FIGURE 2:**
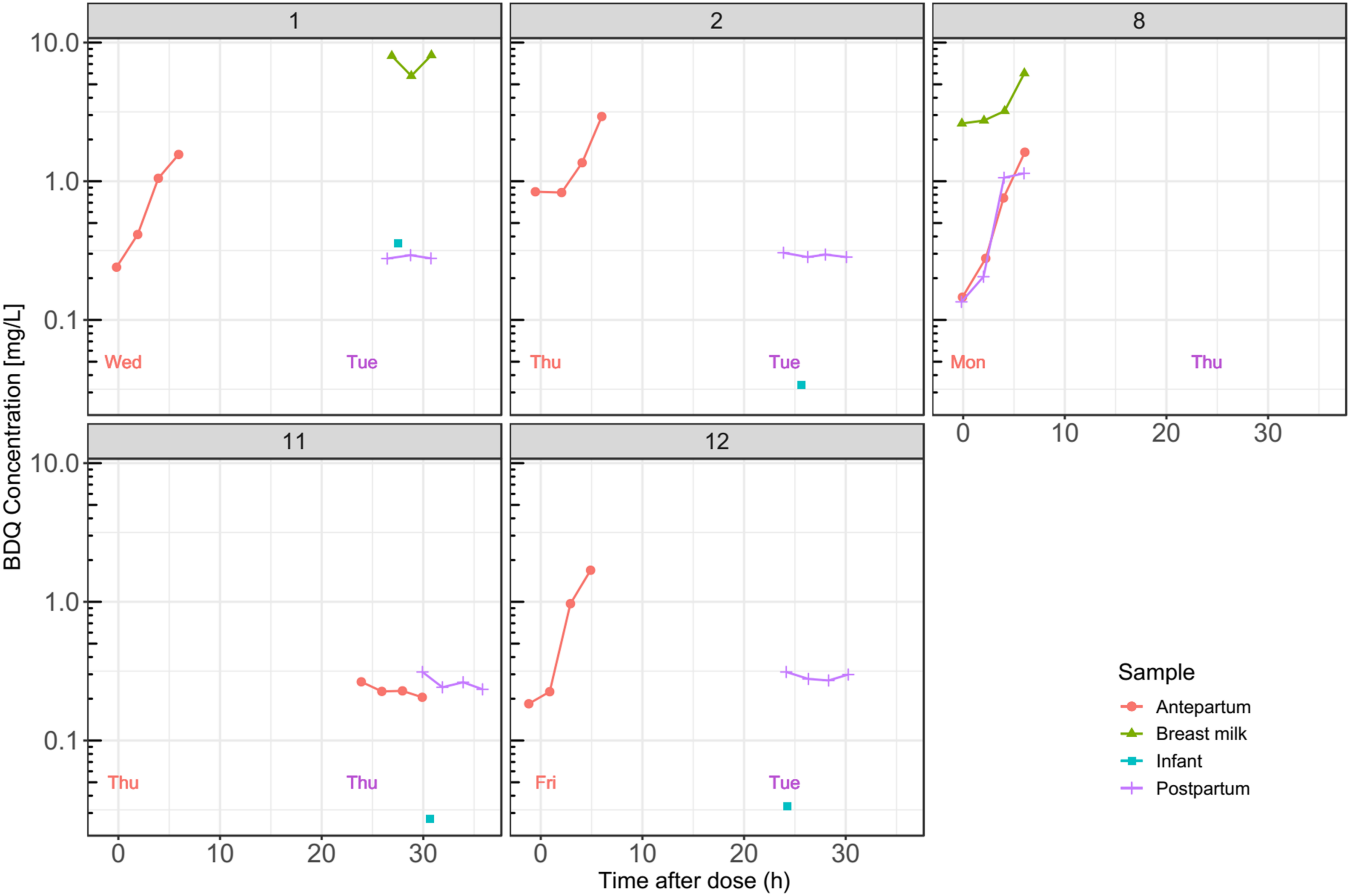
Pharmacokinetics profiles of bedaquiline concentrations, each panel representing a different participant. The red dots and purple crosses represent maternal plasma concentrations ante‐ and postpartum, respectively. The green triangles represent breast milk; the blue squares represent infant plasma concentrations. Bedaquiline was dosed on Monday, Wednesday and Friday, hence the day of the weeks provided in the plot specify if the participant was dosed on the pharmacokinetic visit day

**FIGURE 3:**
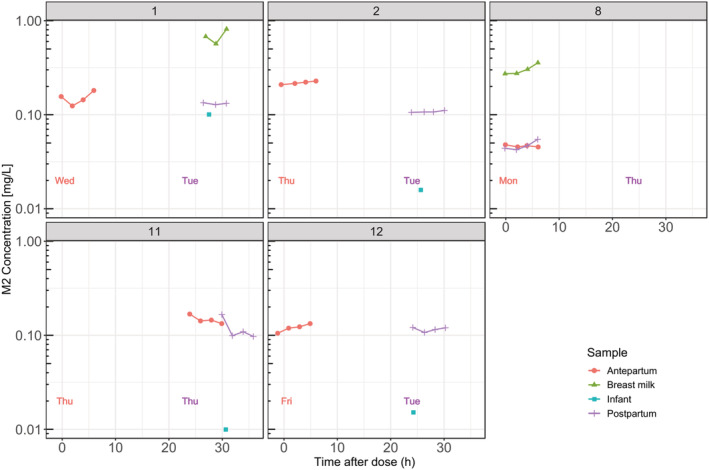
Pharmacokinetics profiles of M2 concentrations, each panel representing a different participant. The red dots and purple crosses represent maternal plasma concentrations ante‐ and postpartum, respectively. The green triangles represent breast milk; the blue squares represent infant plasma concentrations. Bedaquiline was dosed on Monday, Wednesday and Friday, hence the day of the weeks provided in the plot specify if the participant was dosed on the PK visit day

A graphical overview of the infant and breast milk data is provided in Figures 2 and 3, together with the plasma concentrations in the respective mothers. The PK profiles for bedaquiline and M2 are shown: maternal plasma concentrations ante‐ and postpartum; breast milk and infant concentrations. The model estimated an M:P ratio of 13.6 (%relative standard error [RSE]: 10.1) and 4.84 (%RSE: 5.10) for bedaquiline and M2, respectively. The average bedaquiline concentration in the mothers' postpartum PK samples was 0.4 mg/L; using this value the infant bedaquiline dose would be 0.816 mg/kg/d. Similarly, the average maternal postpartum M2 concentration was 0.1, the infant M2 dose would therefore be 0.07 mg/kg/d. In comparison, a 70‐kg individual administered the standard dose of 200 mg bedaquiline 3 times a week would result in approximately 1.22 mg/kg/d dose of bedaquiline. Table S3 displays the breast milk concentrations and their corresponding M:P ratio. Further details of the breast milk concentration model are presented in the appendix. Bedaquiline and M2 concentrations in the infant who was breastfed were similar to maternal plasma concentrations, while for the 3 infants who were not breastfed, bedaquiline and M2 concentrations were detectable but lower than maternal plasma values (see Figures 2 and 3).

## 4 DISCUSSION

To our knowledge, this report is the first description of the exposure of bedaquiline in pregnant women. We found bedaquiline and M2 exposure in pregnant women to be approximately 50% lower than expected in nonpregnant patients.^14^ Although we were underpowered, we found no significant difference between ante‐ and postpartum exposures.

There are several possible reasons for the low bedaquiline exposures we observed in the third trimester. First, increased metabolism of bedaquiline is a possible explanation—pregnancy is known to induce CYP3A4, which is the major route of bedaquiline metabolism.^27^ The increase in CYP3A4 expression would lead to higher clearance and lower bioavailability of bedaquiline, since it is present in both entero‐ and hepatocytes. Second, pregnancy reduces plasma albumin concentrations, to which bedaquiline is highly bound.^28^ The unbound fraction of bedaquiline may therefore increase, subsequently increasing its clearance and tissue distribution. In such a scenario, the total (bound + unbound) concentrations of bedaquiline in plasma would decrease, but this effect could be counter‐balanced by the large unbound fraction, thus maintaining relatively unchanged unbound levels. However, exploration of unbound bedaquiline exposure is required before a recommendation for a dose adjustment can be made. Third, changes in body size (and possibly composition) may have affected bedaquiline disposition, but it is unlikely that the increased weight in pregnancy affected the exposure of bedaquiline as we used allometric scaling to account for this in the model, and changes in body size alone are therefore unlikely to explain the decreased bedaquiline concentrations we observed.

Similarly, we observed lower‐than‐expected bedaquiline levels at the postpartum visit. While it is generally accepted that PK sampling approximately 6 weeks postpartum is a reasonable time‐point to allow the physiological changes related to pregnancy to subside,^29^ there are some limitations in using this timeline as a control when exploring the effect of pregnancy on drugs with a long half‐life such as bedaquiline. Given that the terminal half‐life of bedaquiline is >5 months,^5^ any change in PK parameters may only become apparent on drug exposure after a considerable time, possibly months. Thus, even if most of the pregnancy effects (if any) had reversed in the first weeks after delivery, there may not have been sufficient time for the exposure of bedaquiline to reach a new equilibrium before the scheduled postpartum PK visit. An alternative explanation is that adherence could have decreased in the postpartum period; a systematic review reported poor postpartum adherence in patients on ART.^30^ Subtherapeutic bedaquiline exposures could affect clinical outcomes and increase the risk of selecting for drug resistance.

We observed concerningly high concentrations of bedaquiline in the breast milk samples we analysed, markedly higher than the maternal bedaquiline plasma concentrations, in keeping with the findings of an animal study.^11^ The breastfeeding infant had a plasma bedaquiline concentration similar to maternal plasma (Figure 2), which could have implications for infant safety. In a previous animal study, rat pups that were breastfed to mothers treated with bedaquiline were reported to have low body weight.^11^ In contrast, therapeutic concentrations of bedaquiline in infants (possible with long half‐life drugs, which accumulate slowly, such as bedaquiline) could potentially be protective in infants exposed to RR‐TB, obviating the need for TB preventive therapy. The 3 infants who were not breastfed had subtherapeutic bedaquiline concentrations, probably from transplacental exposure, which could select for drug resistance should the infants develop RR‐TB. A preclinical study in rats treated with bedaquiline also demonstrated placental bedaquiline distribution.^31^

The gestational age at birth of the neonates who had had PK sampling ranged from 33–38 weeks (Table S1). The CYP3A system in the liver and intestinal wall of preterm neonates has lower activity compared with adults, but activity increases with increasing age.^32^ Since bedaquiline is metabolised largely by CYP3A4, the immaturity of the infant CYP3A4 metabolic system may have contributed to the high infant bedaquiline concentrations we observed. Although the World Health Organization recommends all 3 group A drugs including bedaquiline for the treatment of children with MDR‐TB age ≥3 years,^33^ there is a lack of safety data of the use of bedaquiline in children <6 years.^34^ The consequence of the therapeutic bedaquiline concentrations we observed in the breastfeeding infant is unknown, but there are potential implications for infant safety.^11^

The main factors determining the transfer of a drug into breast milk are its physicochemical characteristics (such as lipid solubility and degree of ionisation at different pH conditions) and its plasma PK.^35^ Fat‐soluble drugs like bedaquiline cross lipid‐protein cell membranes easily, hence transferring readily into breast milk.^35^ The ease with which drug molecules cross cellular membranes depends on the drug's degree of ionisation, which may vary in different pH conditions. Weak bases such as bedaquiline (pKa = 8.9)^36^ tend to be slightly less ionised in plasma than in milk. This means that unionised plasma bedaquiline will transfer into breast milk, where it is more likely to be ionised, favouring breast milk accumulation of the drug.^37^ Transfer of drugs into breast milk may also be greater in drugs with a low affinity for maternal plasma proteins, but bedaquiline is highly protein‐bound (>99.9%).^5^ An additional factor is molecular weight, as drugs with low weight (<200 Da) reach breast milk more easily, but the molecular weight of bedaquiline is 555.504 Da.^38^ Drugs that have a long plasma half‐life and therefore accumulate, such as bedaquiline, are prone to transfer into breast milk compared with molecules which are cleared rapidly. The high concentration of bedaquiline in breast milk suggests that the mammary glands could be a clearing site for bedaquiline. Excretion could be significant, since, on average, a baby consumes about 0.15 L/kg/d of breast milk.^24^ Moreover, bedaquiline metabolism in breast tissue cannot be excluded, as there are contradictory reports on the expression of CYP3A4 in human breast tissue.^39^, ^40^, ^41^

Our study has several limitations. First, we did not measure unbound bedaquiline concentrations or albumin levels, so we are unable to conclusively determine if the reasons for the low plasma concentrations observed are related to protein binding. Second, there was a high rate of participant loss to follow up, which limited our sample size, as many participants were unable for logistical reasons, to complete the postpartum PK sampling day. Third, PK sampling was not always performed on a day when bedaquiline was scheduled to be administered (dosing is 3 times a week). Although this was accounted for in our modelling, considering we did not use an adherence measure, the date and time of the last bedaquiline dose was obtained via participant self‐report, which could be unreliable.

We report low exposures of bedaquiline in this series of pregnant women treated for RR‐TB. Future studies should analyse bound and unbound bedaquiline concentrations with an adherence measure to better understand the effect of pregnancy on bedaquiline exposure, and assess whether a different dosing recommendation for bedaquiline in pregnancy is indicated. Bedaquiline appeared to significantly accumulate in breast milk, which could be an exposure risk for breastfeeding babies, and should therefore be investigated further.

## COMPETING INTERESTS

The authors declare no conflicts of interest

## CONTRIBUTORS

R.C. lead the study design and developed the protocol with M.L., contributed to the study analytics and drafted the first version of the manuscript with K.G. K.G. cleaned the data, led the PK modelling under the supervision of P.D. and drafted the first version of the manuscript with R.C. B.M. developed and validated the bedaquiline breast milk assay under the supervision of L.W. L.W. supervised the validation of the breast milk bedaquiline assay and contributed to the manuscript. C.W. contributed to the protocol and provided expert input to the analytics and manuscript. H.M. contributed to the protocol, data collection instruments and manuscript. G.M. contributed to the protocol and manuscript, and provided consultant input during the study. P.D. supervised the PK modelling and contributed to the manuscript. M.L. codrafted the protocol with R.C., supervised participant recruitment and sample collection, and contributed to the manuscript. The authors confirm that the Principal Investigator for this paper is R.C. and that he had direct clinical responsibility for patients.

## Supporting information


**TABLE S1** Additional characteristics of 13 HIV‐positive pregnant women treated for rifampicin‐resistant tuberculosis

**TABLE S2** Final pharmacokinetic parameter estimates for bedaquiline and M2 in breast milk

**TABLE S3** Maternal, breast milk and infant bedaquiline (BDQ) and M2 concentrations in the women with corresponding breast milk samples and the calculated M:P ratio (M:P = milk/maternal plasma) and absolute infant dose.

**FIGURE S1** Pharmacokinetics profile of bedaquiline and M2 of the 2 individuals contributing breast milk samples. Bedaquiline concentrations are plotted in blue and M2 in green. The solid line represents the model‐predicted plasma concentration, while the dashed lines represent the breast milk concentrations. The circles represent the observed breast milk concentrations, while the triangles represent the observed plasma concentrations.

**FIGURE S2** Schematic representation of the PK model of bedaquiline and M2 in plasma and breast milk. The plasma PK model is as reported by Svensson *et at*.^1^ The absorption is described with a series of transit compartments (NN) and mean transit time (MTT) to capture the delay in absorption, and a rate constant ka. Drug transfer between the central and peripheral compartments is defined by intercompartmental clearance Q1 and Q2. Bedaquiline and M2 clearances are denoted by CL and CLM2, respectively. Kmilk is the plasma and breast milk equilibration rate constant and Rmilk and M2_Rmilk are bedaquiline and M2 accumulation ratios, respectively.

## Data Availability

The data that support the findings of this study are available from the corresponding author upon reasonable request.

## Appendix A

### Pharmacokinetic model of breast milk

The characterisation of bedaquiline and M2 concentrations in breast milk was obtained by modelling the plasma and breast milk concentrations in the participants with paired plasma and breast milk samples (the plasma and breast milk bedaquiline and M2 raw concentrations are shown in Table S3 **)**. The modelling procedure comprised 2 steps.

As a first step, we used the published plasma pharmacokinetic (PK) model (1) to describe the individual plasma concentrations around the time when breast milk samples were collected. As referred to in the results section of the main manuscript, the previously published model by Brill et al.^1^ overpredicted the overall concentration of both bedaquiline and M2 in our cohort of patients, as shown in the visual predictive check in Figure 1. However, while at the population level the model was systematically over‐predicting the concentrations, at an individual level, thanks to the between‐subject and ‐occasion random effects, the model was able to fit the plasma concentrations reasonably well in the participants with paired plasma and breast milk concentrations, as shown in Figure S1.

As a second step, we fixed the individual plasma PK parameters and used the model‐predicted plasma concentration profile as an input (forcing function) for the model fitting the breast milk concentrations. This is called a sequential modelling approach, and Zhang et al.^2^ showed that it performs as well as the simultaneous modelling method, which was not feasible in our scenario since the plasma PK model showed a systematic over‐prediction at population level.

To characterise the link between plasma and breast milk concentrations, we used an effect compartment approach,^3^ as shown in the diagram in Figure S2 depicting the structural model. This paradigm describes the concentrations of bedaquiline (and M2) in breast milk as dependent on plasma concentrations, but it assumes no significant transfer of drug between plasma and breast milk (negligible mass transfer), so that the movement of drug into the breast milk compartment does not affect the amount in the central compartment.

The equation describing the concentration in breast milk is:

$$\frac{dC_{\text{milk}}}{\mathrm{dt}}=K_{\text{milk}}\cdot \left(R_{\text{milk}}\cdot C_{\text{plasma}}-C_{\text{milk}}\right)$$

where *C*_*milk*_ is the concentration in breast milk, *C*_*plasma*_ is the plasma concentration, *K*_*milk*_ is the first‐order plasma‐to‐breast milk equilibration rate constant, and *R*_*milk*_ is the accumulation ratio between plasma and breast milk, previously referred to as pseudo‐partition coefficient.^4^, ^5^ *K*_*milk*_ describes the *delay* in the transfer of drugs from plasma to breast milk. It can also be parameterised as a half‐life (*T*_*1/2milk*_ *= In(2)/K*_*milk*_), which can be interpreted as the time required to achieve 50% of the equilibrium target between breast milk and plasma. *R*_*milk*_ is the ratio between the concentrations in breast milk and plasma at equilibrium. Two separate effect compartments were fit, 1 for bedaquiline and the other 1 for M2.

The model parameters are presented in Table S2. The final model did not find any significant difference for *K*_*milk*_ of BDQ and M2, so a single parameter was estimated. The model supported between‐subject variability in bedaquiline *R*_*milk*_ (change in objective function value = 5.05) and only proportional error for both bedaquiline and M2. The model‐predicted profile and the individual PK profile of the plasma and milk concentrations are depicted in Figure S2, showing satisfactory goodness of fit.

The model estimated a bedaquiline milk to plasma accumulation ratio of 13.6 and M2 milk to plasma ratio of 4.84. A single *K*_*milk*_ with a half‐life of 8.15 was estimated for both bedaquiline and M2. However, the large delay in the milk to plasma equilibration might be driven by a single unexpectedly low plasma concentration in the first 6 hours, and hence should be interpreted with caution.

### REFERENCES

1. Brill MJE , Svensson EM , Pandie M , Maartens G , Karlsson MO . Confirming model‐predicted pharmacokinetic interactions between bedaquiline and lopinavir/ritonavir or nevirapine in patients with HIV and drug‐resistant tuberculosis. Int J Antimicrob Agents 2017;49(2):212–7. doi:10.1016/j.ijantimicag.2016.10.020 28038962
2. Zhang L , Beal SL , Sheiner LB . Simultaneous vs. Sequential Analysis for Population PK/PD Data I: Best‐Case Performance. J Pharmacokinet Pharmacodyn 2003 Dec;30(6):387–404. doi:10.1023/B:JOPA.0000012998.04442.1f 15000421
3. Upton R , Mould D. Basic Concepts in Population Modeling, Simulation, and Model‐Based Drug Development: Part 3‐Introduction to Pharmacodynamic Modeling Methods. CPT Pharmacometrics Syst Pharmacol 2014 Jan;3(1):88. doi:10.1038/psp.2013.71 PMC391732024384783
4. Sheiner LB , Stanski DR , Vozeh S , Miller RD , Ham J. Simultaneous modeling of pharmacokinetics and pharmacodynamics: Application to *d* ‐tubocurarine. Clin Pharmacol Ther 1979 Mar;25(3):358–71. doi:10.1002/cpt1979253358 761446
5. Egbelowo O , Sarathy JP , Gausi K , Zimmerman MD , Wang H , Wijnant G‐J , et al. Pharmacokinetics and Target Attainment of SQ109 in Plasma and Human‐Like Tuberculosis Lesions in Rabbits. Antimicrob Agents Chemother 2021 Sep 17;65(9):e0002421. doi:10.1128/AAC.00024-21 34228540PMC8370215
