# Supplementary material for: Bedaquiline exposure in pregnancy and breastfeeding in women with rifampicin‐resistant tuberculosis
Source: Br J Clin Pharmacol. 2022 May 26;88(8):3548–58. doi: 10.1111/bcp.15380 (PMC9296589; doi:10.1111/bcp.15380)
Supplement: Supplementary file 1 — TABLE S1 Additional characteristics of 13 HIV‐positive pregnant women treated for rifampicin‐resistant tuberculosis [file BCP-88-3548-s002.docx]

| **TB treatment regimen** | **ART regimen** | **Gestational age at birth, wks** | **Time on bedaquiline, days** | | **Time from delivery to Postpartum PK, days** | **Breastfeeding status** |
| --- | --- | --- | --- | --- | --- | --- |
|  |  |  | **Antepartum PK** | **Postpartum PK** |  |  |
| LZD^a^, BDQ^b^, hdINH^d^, LFX^l^, CFZ^f^, PZA^g^, EMB^h^ | TDF^m^, FTC^o^, NVP^s^ | 33 | 19 | 81 | 44 | Yes |
| INH, PZA, EMB, BDQ, LVX, Moxy | TDF, FTC, NVP | 38 | 84 | 201 | 46 | No |
| INH, PZA, CFZ, LZD, BDQ, LVX | TDF, FTC, NVP | 37 | 96 | 166 | 48 | No |
| LZD, BDQ, hdINH, LFX, CFZ, PZA, EMB | TDF, FTC, NVP | 37 | 57 |  |  | No |
| INH^e^, PZA, EMB, ETH^i^, CFZ, LZD, BDQ, | TDF, FTC, NVP | 40 | 13 |  |  | No |
| PZA, TRZ^k^, CFZ, LZD, BDQ, DLM^j^ | TDF, FTC, NVP | 39 | 15 |  |  | No |
| LZD, BDQ, hdINH, LFX, CFZ, PZA, EMB | 3TC^l^+RIT^p^+LPV^q^ | 38 | 31 |  |  | No |
| LZD, BDQ, hdINH, LFX, CFZ, PZA, EMB | TDF, FTC, NVP | 40 | 27 | 184 | 44 | Yes |
| LZD, BDQ, hdINH, LFX, CFZ, PZA, EMB | TDF, FTC, NVP | 39 | 17 |  |  | No |
| LZD, BDQ, DLM^c^, LFX, CFZ, PZA, EMB, TRZ, PAS | TDF, FTC, NVP | 39 | 25 |  |  | No |
| LZD, BDQ, DLM, LFX, CFZ, PZA, EMB, TRZ, PAS | TDF, FTC, NVP | 38 | 31 | 143 | 44 | No |
| LZD, BDQ, hdINH, LFX, CFZ, PZA, EMB | DTG^r^, TDF, 3TC | 34 | 35 | 81 | 46 | No |
| LZD, BDQ, hdINH, LFX, CFZ, PZA, EMB | DTG, TDF, 3TC | 36 | 18 |  |  | No |

**Table S1: Additional characteristics of 13 HIV-positive pregnant women treated for rifampicin-resistant tuberculosis**

^a^linezolid; ^b^bedaquiline; ^c^delamanid; ^d^high-dose INH; ^e^isoniazid; ^f^clofazimine, ^g^pyrazinamide, ^h^ethambutol; ^i^ethionamide; ^j^delamanid; ^k^terizidone; ^l^levofloxacin; ^m^tenofovir; ^n^lamivudine; ^o^emtricitabine; ^p^ritonavir; ^q^lopinavir; ^r^dolutegravir, ^s^nevirapine
