# Supplementary material for: Bedaquiline exposure in pregnancy and breastfeeding in women with rifampicin‐resistant tuberculosis
Source: Br J Clin Pharmacol. 2022 May 26;88(8):3548–58. doi: 10.1111/bcp.15380 (PMC9296589; doi:10.1111/bcp.15380)
Supplement: Supplementary file 2 — TABLE S2 Final pharmacokinetic parameter estimates for bedaquiline and M2 in breast milk [file BCP-88-3548-s003.docx]

**Table S2: Final pharmacokinetic parameter estimates for bedaquiline and M2 in breast milk**

| Parameter | Typical Value (%RSE) | Between-Subject Variability ^a^, %CV |
| --- | --- | --- |
| T_1/2milk_ - half-life of delay in plasma to milk equilibration (h) | 8.15 (36.5) |  |
| R_milk_ - BDQ accumulation ratio (.) | 13.6 (10.1) | 10.9 (34.2) |
| M2_R_milk_ - M2 accumulation ratio (.) | 4.84 (5.10) |  |
| BDQ Proportional error (%) | 16.0 (21.1) |  |
| M2 Proportional error (%) | 13.3 (27.5) |  |

^a^ Variability was modelled with log-normal distribution and is presented as an approximate percentage CV
