# Supplementary material for: Bedaquiline exposure in pregnancy and breastfeeding in women with rifampicin‐resistant tuberculosis
Source: Br J Clin Pharmacol. 2022 May 26;88(8):3548–58. doi: 10.1111/bcp.15380 (PMC9296589; doi:10.1111/bcp.15380)
Supplement: Supplementary file 3 — TABLE S3 Maternal, breast milk and infant bedaquiline (BDQ) and M2 concentrations in the women with corresponding breast milk samples and the calculated M:P ratio (M:P = milk/maternal plasma) and absolute infant dose. [file BCP-88-3548-s001.docx]

**Table S3: Maternal, breast milk and infant bedaquiline (BDQ) and M2 concentrations in the women with corresponding breast milk samples and the calculated M:P ratio (M:P= milk/maternal plasma) and absolute infant dose.**

| ID | TAD* (hours) | BDQ maternal plasma (mg/L) | M2 maternal plasma (mg/L) | BDQ breast milk (mg/L) | M2 breast milk (mg/L) | BDQ M:P | M2 M:P |
| --- | --- | --- | --- | --- | --- | --- | --- |
| 1 | 26.45 | 0.277 | 0.134 | 8.02 | 0.679 | 29.0 | 5.07 |
| 1 | 28.75 | 0.293 | 0.128 | 5.75 | 0.568 | 19.6 | 4.44 |
| 1 | 30.75 | 0.278 | 0.132 | 8.11 | 0.814 | 29.2 | 6.17 |
| 8 | 47.83 | 0.135 | 0.044 | 2.61 | 0.273 | 19.3 | 6.20 |
| 8 | 2 | 0.205 | 0.0425 | 2.74 | 0.275 | 13.4 | 6.47 |
| 8 | 4.01 | 1.06 | 0.0467 | 3.21 | 0.304 | 3.02 | 6.51 |
| 8 | 5.98 | 1.14 | 0.0547 | 6.01 | 0.357 | 5.27 | 6.523 |

* TAD: Time after the last bedaquiline dose.
