# Supplementary material for: Bedaquiline exposure in pregnancy and breastfeeding in women with rifampicin‐resistant tuberculosis
Source: Br J Clin Pharmacol. 2022 May 26;88(8):3548–58. doi: 10.1111/bcp.15380 (PMC9296589; doi:10.1111/bcp.15380)
Supplement: Supplementary file 4 — FIGURE S1 Pharmacokinetics profile of bedaquiline and M2 of the 2 individuals contributing breast milk samples. Bedaquiline concentrations are plotted in blue and M2 in green. The solid line represents the model‐predicted plasma concentration, while the dashed lines represent the breast milk concentrations. The circles represent the observed breast milk concentrations, while the triangles represent the observed plasma concentrations. [file BCP-88-3548-s004.pdf]

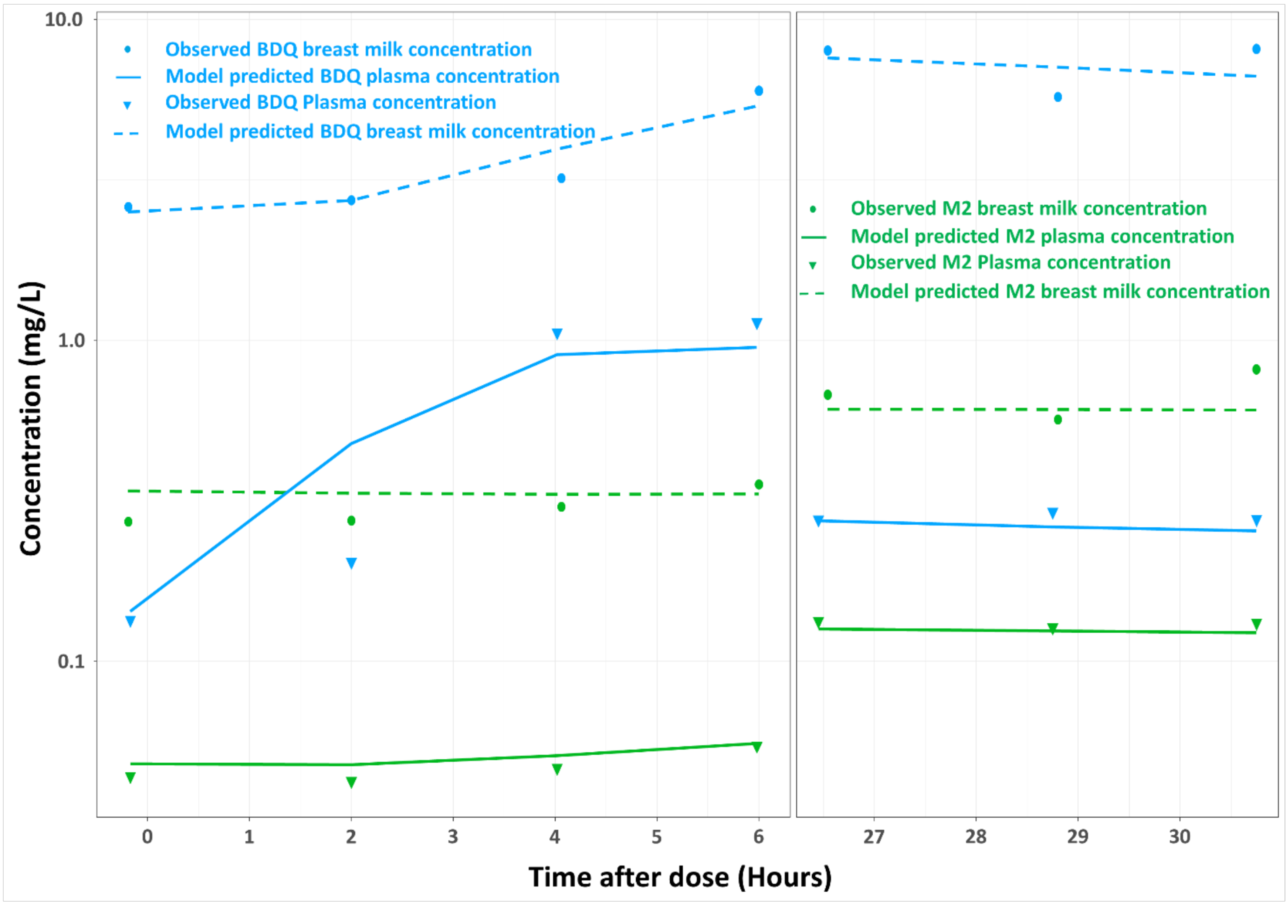

Figure S1: Pharmacokinetics profile of bedaquiline and M2 of the two individuals contributing breast milk samples. Bedaquiline concentrations are plotted in blue and M2 in green. The solid line represents the model-predicted plasma concentration, while the dashed lines represent the breast milk concentrations. The circles represent the observed breast milk concentrations, while the triangles represent the observed plasma concentrations.
