# Supplementary material for: Bedaquiline exposure in pregnancy and breastfeeding in women with rifampicin‐resistant tuberculosis
Source: Br J Clin Pharmacol. 2022 May 26;88(8):3548–58. doi: 10.1111/bcp.15380 (PMC9296589; doi:10.1111/bcp.15380)
Supplement: Supplementary file 5 — FIGURE S2 Schematic representation of the PK model of bedaquiline and M2 in plasma and breast milk. The plasma PK model is as reported by Svensson et at.1 The absorption is described with a series of transit compartments (NN) and mean transit time (MTT) to capture the delay in absorption, and a rate constant ka. Drug transfer between the central and peripheral compartments is defined by intercompartmental clearance Q1 and Q2. Bedaquiline and M2 clearances are denoted by CL and CLM2, respectively. Kmilk is the plasma and breast milk equilibration rate constant and Rmilk and M2_Rmilk are bedaquiline and M2 accumulation ratios, respectively. [file BCP-88-3548-s005.pdf]

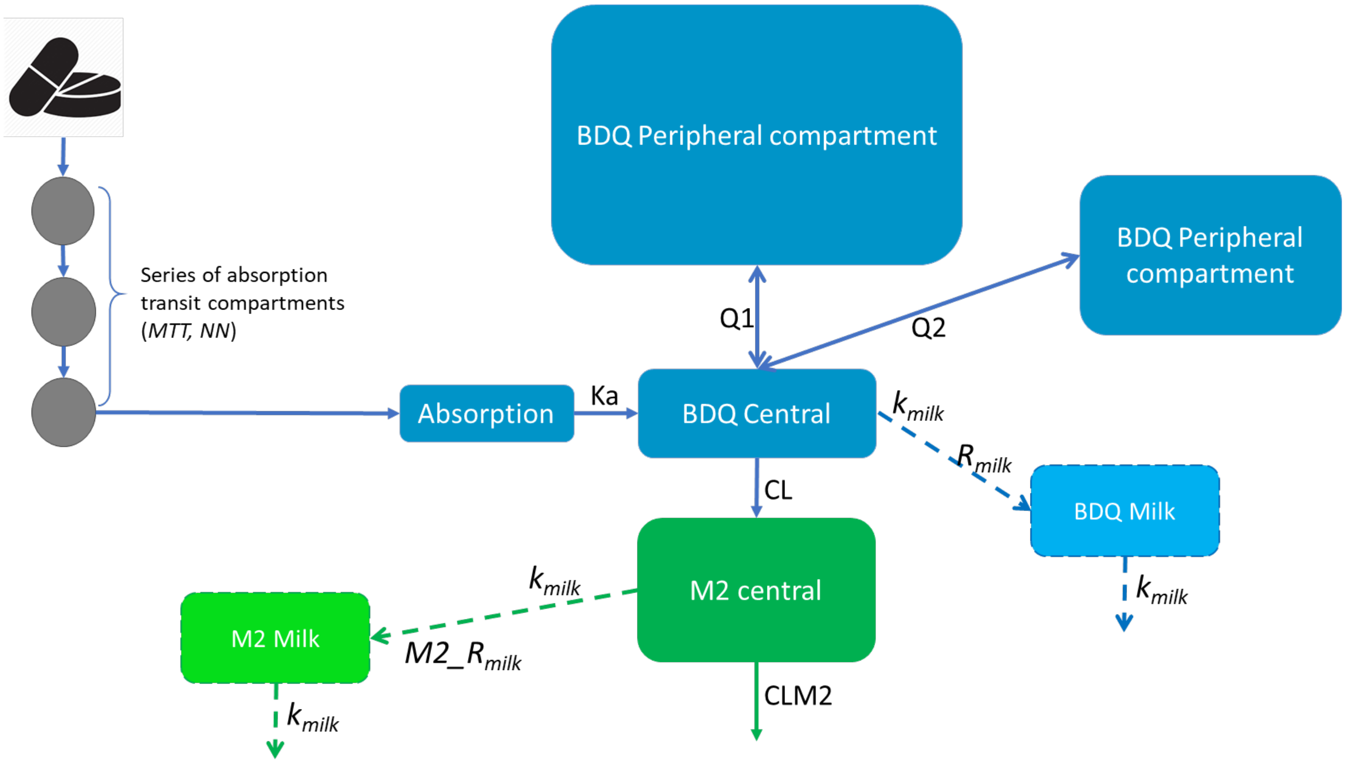

Figure S2: Schematic representation of the PK model of bedaquiline and M2 in plasma and breast milk. The plasma PK model is as reported by Svensson et at. 2016 (1). The absorption is described with a series of transit-compartments (NN) and mean transit time (MTT) to capture the delay in absorption, and a rate constant ka. Drug transfer between the central and peripheral compartments is defined by intercompartmental clearance Q1 and Q2. Bedaquiline and M2 clearances are denoted by CL and CLM2, respectively. K_{milk} is the plasma and breast milk equilibration rate constant and R_{milk} and M2_R_{milk} are bedaquiline and M2 accumulation ratios, respectively.
